# The role of education in the association between race/ethnicity/nativity, cognitive impairment, and dementia among older adults in the United States

**DOI:** 10.4054/DemRes.2018.38.6

**Published:** 2018-01-11

**Authors:** Marc A. Garcia, Joseph Saenz, Brian Downer, Rebeca Wong

**Affiliations:** 1 Sealy Center on Aging (SCOA), University of Texas Medical Branch, USA. marcagar@utmb.edu.; 2 Davis School of Gerontology, University of Southern California, USA.; 3 Division of Rehabilitation Sciences, School of Health Professions, University of Texas Medical Branch, USA.; 4 Department of Preventive Medicine and Community Health, University of Texas Medical Branch, USA.

## Abstract

**BACKGROUND:**

Older Black and Hispanic adults are more likely to be cognitively impaired than older White adults. Disadvantages in educational achievement for minority and immigrant populations may contribute to disparities in cognitive impairment.

**OBJECTIVE:**

Examine the role of education in racial/ethnic and nativity differences in cognitive impairment/no dementia (CIND) and dementia among older US adults.

**METHODS:**

Data comes from the 2012 Health and Retirement Study. A total of 19,099 participants aged ≥50 were included in the analysis. Participants were categorized as having normal cognition, CIND, or dementia based on the Telephone Interview for Cognitive Status (TICS) or questions from a proxy interview. We document age and educational differences in cognitive status among White, Black, US-born Hispanic, and foreign-born Hispanic adults by sex. Logistic regression is used to quantify the association between race/ethnicity/nativity, education, and cognitive status by sex.

**RESULTS:**

Among women, foreign-born Hispanics have higher odds of CIND and dementia than Whites. For men, Blacks have higher odds for CIND and dementia compared to Whites. The higher odds for CIND and dementia across race/ethnic and nativity groups was reduced after controlling for years of education but remained statistically significant for older Black and US-born Hispanic adults. Controlling for education reduces the odds for CIND (women and men) and dementia (men) among foreign-born Hispanics to nonsignificance.

**CONTRIBUTION:**

These results highlight the importance of education in CIND and dementia, particularly among foreign-born Hispanics. Addressing inequalities in education can contribute to reducing racial/ethnic/nativity disparities in CIND and dementia for older adults.

## 1. Introduction

Extensive research has been conducted on racial and ethnic disparities in cognitive functioning in the United States. These findings indicate that older non-Hispanic Blacks (hereafter, Blacks) and Hispanics have lower cognitive performance than non-Hispanic Whites (hereafter, Whites) on measures of memory (Masel and Peek 2009), executive functioning (Early et al. 2013), and global cognition (Díaz-Venegas et al. 2016). Blacks and Hispanics are also more likely to have cognitive impairment, and spend a larger proportion of their remaining years after age 50 with cognitive impairment/no dementia (CIND) and dementia than Whites (Alzheimer’s Association 2010; Garcia et al. 2017a; Langa et al. 2017). These racial/ethnic disparities in cognitive functioning have been attributed to several factors including a higher prevalence of chronic health conditions associated with an increased risk for dementia (Mayeda et al. 2015), poor educational quality in childhood (Crowe et al. 2013), and low literacy (Mehta et al. 2004).

Prior research has consistently found that higher educational attainment is associated with better performance on measures of cognitive functioning (Alley, Suthers, and Crimmins 2007) and decreased risk for dementia (Caamaño-Isorna et al. 2006). Hispanics, in particular, have lower educational attainment compared to Whites. In 2014, nearly 90% of Whites aged 55 and older reported having at least a high school level of education, compared to 80% for Blacks and 59% for Hispanics of comparable age (United States Census Bureau 2014). Racial and ethnic disparities in cognitive functioning may also be attributed to disadvantages in educational achievement for minority and immigrant populations. Controlling for differences in education has been shown to reduce disparities in cognition between Whites, Blacks, and Hispanics (Schwartz et al. 2004; Sisco et al. 2015). For example, Yaffe and colleagues showed the increased risk for dementia among older Blacks compared to Whites was no longer statistically significant after controlling for a composite measure of socioeconomic status that included educational attainment, income, financial adequacy, and literacy level (Yaffe et al. 2013).

Despite considerable research into racial/ethnic disparities in cognitive functioning among older adults and the role of education in explaining these disparities, less scholarship has examined if the likelihood for CIND and dementia among older Hispanics varies by nativity status (i.e., US-born vs. foreign-born) compared to Whites. Prior research documents the risk for cognitive impairment, rates of cognitive decline, and proportion of years after age 65 lived with cognitive impairment vary by nativity status among older Hispanics (Downer et al. 2017; Garcia et al. 2017b; Garcia et al. forthcoming; Hill, Angel, and Balistreri 2012; Hill et al. 2012). However, these analyses only included US-born and foreign-born Hispanics of Mexican origin residing in the Southwest United States, which prevented racial/ethnic and nativity comparisons in cognitive status with older White and Black adults.

Recent research from the Health and Retirement Study (HRS) showed that the increased odds for prevalent cognitive impairment among US-born and foreign-born Mexican Americans was reduced and no longer statistically significant compared to Whites after controlling for individual social and economic factors, neighborhood socioeconomic status, and immigrant homogeneity. In addition, this study documented an immigrant advantage among foreign-born Mexican Americans in the incidence of cognitive impairment compared to Whites, independent of individual and neighborhood characteristics. However, this analysis did not differentiate by sex or between CIND and dementia when defining cognitive impairment (Weden et al. 2017).

The relationship between nativity, cognitive functioning, cognitive impairment, and cognitive life expectancies among older Hispanics has been shown to differ between men and women (Downer et al. 2017; Garcia et al. 2017a; Garcia et al. 2017b; Garcia et al. forthcoming; Hill, Angel, and Balistreri 2012; Hill et al. 2012). Differentiating between CIND and dementia is important because CIND is a less severe stage of cognitive impairment. The present analysis examines the role of education in racial/ethnic and nativity differences for CIND and dementia. This analysis extends previous research by (1) distinguishing different cognitive statuses, (2) differentiating between Hispanics by nativity, and (3) stratifying by sex.

## 2. Data and methods

This analysis is based on data from the 2012 Health and Retirement Study (HRS 2011). We use the harmonized version of the RAND HRS Version O Data File (RAND 2015) to assess the association between education and the odds for CIND and dementia among White, Black, and US-born and foreign-born Hispanic adults ages 50 and older in the United States. Respondents missing information on education and who identified as ‘other’ are omitted from the analysis. The final analytic sample includes 12,762 Whites, 3,715 Blacks, 992 US-born Hispanics, and 1,630 foreign-born Hispanics for a total of 19,099 participants.

The cognitive functioning of HRS participants able to complete a direct interview is evaluated using a modified version of the Telephone Interview for Cognitive Status (TICS-M) (Brandt, Spencer, and Folstein 1988). The TICS-M assesses cognitive functioning in learning (immediate recall of 10-word list, 10 points), memory (delayed recall of 10-word list, 10 points), working memory (serial seven subtraction, 5 points), and attention (counting backward from 20–11, 2 points) (Crimmins et al. 2011). A total score for the TICS-M is obtained by calculating the sum of the individual domains. The range of possible scores is 0–27 points with higher scores indicating better cognitive functioning. We used the TICS-M as HRS participants younger than 65 years of age are not given the orientation or naming items that are included in the full cognitive assessment (Crimmins et al. 2011). Following previous research, we used cutoffs created to classify participants as having normal cognition (12–27 points), CIND (7–11 points), and dementia (0–6 points) (Crimmins et al. 2011). These cutoffs were created by HRS investigators so the frequency of cognitive states in the HRS matched what was estimated in the Aging Demographics and Memory Study (Crimmins et al. 2011), which is a substudy of the HRS who received an in-depth in-home neuropsychological exam.

The cognitive status of HRS participants who were unable to complete a direct interview is categorized using questions from a proxy interview (Crimmins et al. 2011): (1) proxy-reported memory ability (0 points [excellent] – 4 points [poor]); (2) number of limitations in five instrumental activities of daily living (managing money, taking medication, preparing meals, using a telephone, and shopping for groceries; score 0–5 points); and (3) interviewer assessment of difficulty completing the interview due to respondent’s cognitive limitations (0–2 points). The overall score was used to classify proxy respondents with dementia (6 points or higher), CIND (3–5 points), or normal cognition (0–2 points).

Sociodemographic variables in the analysis include race/ethnicity, nativity, sex, age, and education. Race/ethnicity and nativity are self-reported. We include Whites, Blacks, and US-born and foreign-born Hispanics. Sex corresponds to whether the respondent identifies as female or male. Age is a continuous variable. We measure education as completed years of formal education.

In the descriptive analysis, comparisons across age, education, and cognitive status were conducted using *X*^*2*^ and *t*-tests to assess race/ethnicity and nativity differentials by sex. For the multivariate models, we used logistic regression to quantify the association between race/ethnicity/nativity, education, and cognitive status by sex. All models were fit separately for males and females to account for well-known sex differences in aging, including a higher lifetime risk for dementia and lower levels of education among women compared to men (Chêne et al. 2015; Ott et al. 1998). We use sampling weights provided by the HRS to adjust for nonresponse and the complex design of the survey.

## 3. Results

Table 1 presents sociodemographic characteristics for the study sample. White respondents are significantly older, more educated, and less likely to be classified as CIND or dementia than minority and immigrant groups, regardless of sex. In addition, females are older than their male counterparts and report fewer years of education across race/ethnicity and nativity (with the exception of education for Blacks).

Table 2 presents cognitive status characteristics by race/ethnicity/nativity and sex. Overall, White adults were more likely to have normal cognitive status at older ages than minority and immigrant groups. In addition, the mean age for CIND and dementia is significantly lower among older Whites than Blacks and Hispanics. Furthermore, older Whites have significantly more years of education across all cognitive categories compared to Blacks and Hispanics, regardless of sex.

Table 3 presents results from separate logistic regression models for CIND and dementia. For each regression model, the reference category is cognitively normal. Models 1 and 3 are base models that examine race/ethnic/nativity differentials controlling for age. Models 2 and 4 add education. The results in Panel A, for females, show that race/ethnicity/nativity and older age are associated with CIND and dementia. In Model 1, Black, US-born Hispanic, and foreign-born Hispanic women have higher odds (4.8, 4.1, and 5.4, respectively) of being classified as CIND than White women. In Model 3, Blacks, US-born Hispanics, and foreign-born Hispanics have higher odds (5.9, 6.4, and 8.9, respectively) of being classified as having dementia than White women, independent of age. Although adding education attenuates disparities in CIND (Model 2) and dementia (Model 4) among women, the higher odds of CIND and dementia for minority and immigrant groups remain statistically significant (with the exception of CIND for foreign-born Hispanic women).

Among males (Panel B), a different pattern emerges. All minority and immigrant men have higher odds of CIND and dementia than White men, but Blacks exhibit 4.1 times higher odds while both US-born Hispanics and foreign-born Hispanics exhibit 3.1 times higher odds for CIND (Model 1). Similarly, in Model 3, Black, US-born Hispanic, and foreign-born Hispanic men have higher odds (8.3, 6.4, and 3.4, respectively) of being classified as having dementia compared to White men. When we include education, the odds for CIND (Model 2) and dementia (Model 4) are reduced among Blacks and US-born Hispanics; for foreign-born Hispanic males the difference for CIND and dementia is no longer significant.

Models 2 and 4 for both sexes illustrate the impact of lower levels of education on cognitive status for older Blacks and Hispanics relative to Whites. Educational disadvantages among minority and immigrant groups contribute to the higher odds for CIND and dementia. These findings suggest that the increased odds for CIND and dementia among minority and immigrant populations relative to Whites would be reduced, but still present for some groups if years of education were equal across populations.

## 4. Discussion and conclusion

Our findings call attention to the importance of race/ethnicity/nativity and education when assessing the odds for CIND and dementia among older adults in the United States. First, we provide evidence that years of education accounts for a large proportion of the association between race/ethnicity/nativity, CIND, and dementia among Blacks, US-born Hispanics, and foreign-born Hispanics. Second, the role of education appears to be stronger for foreign-born Hispanics compared to Blacks and US-born Hispanics.

It is important to interpret our findings in the context of previous research using the Health and Retirement Study. For example, analyses of data from the 2006 HRS has shown that among adults 55 years and older, Blacks were two to three times, and Hispanics two times, more likely to be cognitively impaired than Whites (Alzheimer’s Association 2010). Though these disparities varied by age group, with larger racial/ethnic differences among adults aged 55–64 (four times more likely for Blacks and three times more likely for Hispanics), than adults 85 and older (two times more likely for Blacks and 1.6 times more likely for Hispanics, respectively) (Alzheimer’s Association 2010). Our findings for the odds of CIND and dementia for Blacks and Hispanics after adjusting for educational attainment are similar to those reported by the Alzheimer’s Association (2010). However, our odds ratios for dementia, particularly for Blacks, are considerably higher than what was reported in a recent study using 2000 and 2012 HRS data (Langa et al. 2017). These differences may be due to several factors. First, Langa and colleagues (2017) combined cognitively normal and CIND categories to create a dichotomous variable. Thus, participants with CIND were included in the reference category. Second, the authors control for net worth as an additional measure of socioeconomic status, which may have reduced the association between race/ethnicity and dementia. Finally, Langa and colleagues (2017) did not stratify by nativity or sex, which may also have contributed to the different findings.

We acknowledge that net worth may contribute to disparities in CIND and dementia. Though, previous findings suggest the increased risk for dementia associated with low income is reduced and no longer statistically significant after controlling for education (Evans et al. 1997). Thus, controlling for income may not have a considerable impact on our results.

In addition, the racial/ethnic and nativity differences reported above may be due in part to temporal trends. Using longitudinal data can help tease out age and cohort effects by comparing data from different time points (Yang and Land 2013). However, recent longitudinal findings in the HRS show no significant differences in the increased odds for prevalent cognitive impairment among US-born and foreign-born Mexican Americans independent of individual social and economic factors (Weden et al. 2017), which are consistent with our findings. Furthermore, this study documents an immigrant advantage in the incidence of cognitive impairment among foreign-born Mexican Americans relative to Whites, consistent with the healthy immigrant hypothesis (Weden et al. 2017). Positive health selection that contributes to longer life expectancies and lower mortality may also contribute to a reduced risk for cognitive impairment and slower cognitive decline among foreign-born Hispanics. For instance, Hill et al. (2012) found mid-life (age 20–49 years) immigrants from Mexico had higher levels of cognitive functioning and slower rates of cognitive decline than their US-born co-ethnics. Furthermore, this study documented midlife migrant males were able to maintain higher cognitive function for a longer period of time compared to midlife migrant females, which is also consistent with the health immigrant effect (Hill et al. 2012).

Further research is needed to shed light on the cognitive benefits of formal education among different groups who most likely received vastly different qualities of formal education. Furthermore, it is possible that different effects of formal years of education for particular groups imply that informal education plays an important supplementary role. Moreover, additional research is needed to deepen our understanding of racial/ethnic and nativity disparities in cognition beyond educational attainment. The CIND and dementia differences we documented above have important policy implications. Educational attainment of Whites remains considerably higher than that of Blacks and Hispanics due to social and economic disadvantages experienced by minority populations (Gamoran 2001). Social policy specifically aimed at increasing educational attainment and quality for minority and immigration populations can potentially have major impacts on reducing or eliminating future disparities in adult CIND and dementia. Our analysis should help advance a social policy agenda aimed toward the eventual closing of cognitive disparities among minority and immigrant groups of older adults in the United States.

## Figures and Tables

**Table 1:** Sociodemographic characteristics by race/ethnicity/nativity and sex

	Whites	Blacks	US-born Hispanics	Foreign-born Hispanics
**Panel A: Females**				
Age (SD)	66.9 (10.5)	64.9 (9.9) ***	64.9 (10.1) ***	64.7 (9.5) ***
Education (SD)	13.5 (2.4)	12.6 (2.8) ***	11.2 (3.5) ***	8.1 (4.8) ***
Cognitive status				
Normal	86.2	64.1 ***	66.4 ***	60.7 ***
CIND	10.5	27.1 ***	23.4 ***	28.4 ***
Dementia	3.3	8.8 ***	10.1 ***	10.9 ***
N	7,270	2,310	566	932
**Panel B: Males**				
Age (SD)	65.7 (9.7)	63.7 (8.9) ***	64.3 (8.7) ***	63.0 (9.1) ***
Education (SD)	13.8 (2.6)	12.4 (2.9) ***	11.9 (3.4) ***	8.5 (5.1) ***
Cognitive status				
Normal	85.3	63.0 ***	67.8 ***	71.0 ***
CIND	12.0	27.8 ***	23.5 ***	23.9 ***
Dementia	2.8	9.2 ***	8.6 ***	5.2 **
N	5,492	1,405	426	698

*Source*: Health and Retirement Study 2012 (N=19,099).

*Note*: Unweighted Ns; weighted percentages and means. Reference category is Whites.

** p<.01;

*** p<.001

**Table 2:** Cognitive status characteristics by race/ethnicity/nativity and sex

	Whites	Blacks	US-born Hispanics	Foreign-born Hispanics
**Panel A: Females**				
**Mean age (SD)**				
Cognitive Status				
Normal	65.4 (9.6)	62.8 (8.1) ***	62.5 (7.8) ***	62.6 (8.1) ***
CIND	74.5 (11.4)	66.1 (10.2) ***	66.1 (10.1) ***	66.0 (9.5) ***
Dementia	80.6 (11.0)	76.5 (12.1) ***	78.5 (12.1) **	73.1 (11.6) ***
**Mean education (SD)**				
Cognitive Status				
Normal	13.7 (2.3)	13.4 (2.3) **	12.3 (2.8) ***	9.6 (4.4) ***
CIND	12.1 (2.4)	11.5 (2.7) **	9.9 (3.4) ***	6.0 (4.6) ***
Dementia	11.5 (3.0)	10.1 (3.3) ***	7.1 (3.9) ***	5.2 (4.0) ***
**Panel B: Males**				
**Mean age (SD)**				
Cognitive Status				
Normal	64.5 (8.9)	61.7 (7.2) ***	62.6 (7.1) ***	61.9 (8.2) ***
CIND	71.4 (11.1)	65.1 (9.3) ***	65.9 (8.9) ***	64.2 (9.3) ***
Dementia	78.8 (10.3)	73.0 (11.2) ***	73.8 (11.8) ***	72.4 (13.4) ***
**Mean education (SD)**				
Cognitive Status				
Normal	14.1 (2.3)	13.2 (2.6) ***	12.7 (2.9) ***	9.6 (5.0) ***
CIND	12.1 (2.7)	11.6 (2.4) **	10.5 (3.1) ***	6.1 (4.6) ***
Dementia	11.5 (3.6)	9.2 (3.6) ***	9.0 (4.9) ***	4.4 (4.3) ***

*Source*: Health and Retirement Study 2012 (N=19,099).

*Note*: Reference category is Whites.

** p<.01;

*** p<.001.

**Table 3:** Odds ratios from logistic regression for CIND and dementia by race/ethnicity/nativity and sex

Predictor variables	CIND		Dementia	
	Model 1	Model 2	Model 3	Model 4
**Panel A: Females**				
Blacks	4.82 ***	4.16 ***	5.86 ***	4.75 ***
US-born Hispanics	4.08 ***	2.54 ***	6.35 ***	2.85 ***
Foreign-born Hispanics	5.43 ***	1.36	8.92 ***	1.71 *
Age	1.07 ***	1.06 ***	1.14 ***	1.13 ***
Years of Education		0.78 ***		0.75 ***
N	9,743	9,743	8,541	8,541
**Panel B: Males**				
Blacks	4.12 ***	3.27 ***	8.26 ***	5.21 ***
US-born Hispanics	3.05 ***	2.01 ***	6.37 ***	3.45 ***
Foreign-born Hispanics	3.11 ***	0.70	3.42 ***	0.55
Age	1.07 ***	1.06 ***	1.14 ***	1.13 ***
Years of Education		0.78 ***		0.76 ***
N	7,301	7,301	6,309	6,309

*Source*: Health and Retirement Study 2012 (N=19,099).

*Note*: Reference category is Whites.

* p<.05;

*** p<.001.
